# Soy versus whey protein bars: Effects on exercise training impact on lean body mass and antioxidant status

**DOI:** 10.1186/1475-2891-3-22

**Published:** 2004-12-08

**Authors:** Erin C Brown, Robert A DiSilvestro, Ari Babaknia, Steven T Devor

**Affiliations:** 1 Department of Sport & Exercise Sciences, The Ohio State University, Columbus, Ohio, USA; 2 Department of Human Nutrition, The Ohio State University, Columbus, Ohio, USA; 3 DrSoy Inc., Irvine, California, USA

## Abstract

**Background:**

Although soy protein may have many health benefits derived from its associated antioxidants, many male exercisers avoid soy protein. This is due partly to a popular, but untested notion that in males, soy is inferior to whey in promoting muscle weight gain. This study provided a direct comparison between a soy product and a whey product.

**Methods:**

Lean body mass gain was examined in males from a university weight training class given daily servings of micronutrient-fortified protein bars containing soy or whey protein (33 g protein/day, 9 weeks, n = 9 for each protein treatment group). Training used workouts with fairly low repetition numbers per set. A control group from the class (N = 9) did the training, but did not consume either type protein bar.

**Results:**

Both the soy and whey treatment groups showed a gain in lean body mass, but the training-only group did not. The whey and training only groups, but not the soy group, showed a potentially deleterious post-training effect on two antioxidant-related related parameters.

**Conclusions:**

Soy and whey protein bar products both promoted exercise training-induced lean body mass gain, but the soy had the added benefit of preserving two aspects of antioxidant function.

## Background

Many male exercisers avoid soy protein because there is a perception that it is inferior to proteins like whey for supporting lean boss mass gain. This perception persists even though there are no studies comparing whey and soy for effects on lean body mass gain. Soy may actually help promote lean body mass gain by the antioxidants associated with soy protein. Antioxidants are agents, either consumed in the diet or made by the body, which work against molecular damage due to oxidant reactions caused by free radicals, which are reactive molecules with an unpaired electron [1]. Soy protein isolate contains a mixture of antioxidants including isoflavones, saponins, and copper, a component of a number of antioxidant enzymes [2]. Body free radical production seems to be particularly high during exercise, and the resulting oxidant stress appears to contribute to muscle damage and fatigue [3]. This damage and fatigue could conceivably limit progress in exercise training by slowing muscle recovery between exercise workouts. This could limit lean body mass gain during an exercise program.

If soy protein can promote lean body mass gain at least as well as whey, there may be one advantage to consuming soy protein. Soy protein contains antioxidants which may not only help with lean body mass gain, but which can also promote other aspects of health. Antioxidant actions are thought to work against the onset and severity of many diseases and health problems [1]. This may be particularly important during exercise training, which in some cases, depletes antioxidant capacities and/or increases oxidant stress [i.e. [4,5]]. This may explain why high degrees of chronic exercise can be detrimental. For example, some athletes show increases in histochemical muscle lesions as well as high cancer mortality, which have been linked to prolonged periods of exercise [6,7]. However, this area has been controversial since some studies suggest that long term exercise training produce body adaptations which increase antioxidant defenses [i.e. [8,9]]. Either way, soy protein antioxidants could conceivably exert beneficial effects during exercise training, either by restricting antioxidant depletion or by enhancing antioxidant capacity increases.

The present study compared a soy protein product to a whey protein product in subjects undergoing a 9 week weight training program. Subjects were evaluated for lean body mass gain and changes in antioxidant status. The latter was done using one measurement of a component of antioxidant capacity and one for a component of oxidant stress. The former was based on an assay called plasma antioxidant status which assesses the ability to scavenge certain chemically generated radicals. The oxidant stress parameter was plasma myeloperoxidase, a measure of neutrophil activation, which is associated with increased secretion of superoxide radical [1].

## Methods

### Subjects

This study was approved by the Human Subjects Review Committee for Biomedical Sciences at The Ohio State University. All subjects signed an informed consent form. Male subjects, aged 19–25, were recruited from the Sport, Fitness and Health Program courses at The Ohio State University to participate in the present 9-week study. All subjects were considered experienced weightlifters with at least 1 year or more experience in strength training, which was confirmed by a questionnaire. Subjects were reported to be non-smokers, non-vegetarians, not currently taking supplements of any kind, and having no major health problems (i.e., diabetes, cardiovascular disease, etc.). All subjects had a body mass index (BMI) of less than 30.

### Strength Training Program

At the start of the study, each subject was put on a common strength training program to strictly follow for the duration of the 9 week study. Subjects were given either workout 1 or workout 2. The two workouts were identical with the exception of exercise order and were designed to prevent subjects in the strength training classes from having to perform the same exercises at the same time. Midway through the program, subjects with workout 1 were given workout 2 and vice versa in order to maintain consistency.

The strength training protocol was 3 sets of 4–6 repetitions for 14 exercises so that strength was the variable being maximized. The following exercises were performed to work all major muscle groups: 1) chest press; 2) chest fly; 3) incline press; 4) lat pull-down; 5) seated row; 6) military press; 7) lateral raise; 8) preacher curl; 9) bicep curl; 10) supine tricep extension; 11) seated tricep extension; 12) leg press; 13) calf raise; and 14) abdominal crunches.

### Protein Treatments

Subjects were randomly assigned in a double-blind manner to either a soy, whey, or control group. The controls did the exercise program but did not consume a protein product (n = 9/each group). The soy protein product was DrSoy^® ^Bars, which contained 11 grams of protein and an assortment of micronutrients. The whey bars were made using the same recipe as the DrSoy^® ^Bars except that whey protein was substituted for soy protein. The products were supplied to study personnel in plain wrappers with different colors for each product. The color code was unknown to the subjects and study personnel who were in contact with the subjects. Each subject was instructed to consume 3 bars per day for the 9-week training period. This was in addition to the subjects' self-selected diet. Subjects were instructed not to change eating patterns during the course of the study. The time of the day when the bars were consumed was recorded daily in the subject's fitness log so that compliance could be monitored.

### Measurements

Lean body mass was analyzed by hydrostatic weighing. Each subject performed at least 3 efforts and an average reading was taken. Blood was drawn into heparin tubes before and after the 9 week treatment period on a day when the subjects did not exercise. Blood was spun at 3000 × g and the plasma was stored at -70°C until analysis. Unfortunately, a problem during blood processing made some plasma samples unavailable for analysis. Plasma was analyzed for free radical scavenging capacity using the Total Antioxidant Status Assay Kit from Calbiochem-Novachem Corp. (San Diego, CA). Plasma myeloperoxidase was analyzed using an ELISA kit from Calbiochem-Novachem.

### Statistical analysis

Statistical analysis was done by the Jump 3.1 program (SAS Institute, Cary, NC), with significance at p < 0.05. For each parameter and treatment group, values prior to the 9 week treatment were compared to values after treatment by paired, 2-tailed Student's t-test. In addition, for lean body mass, the changes in values for soy treatment were compared to the change in values for the other two groups by Tukey test.

## Results

**Figure 1:**
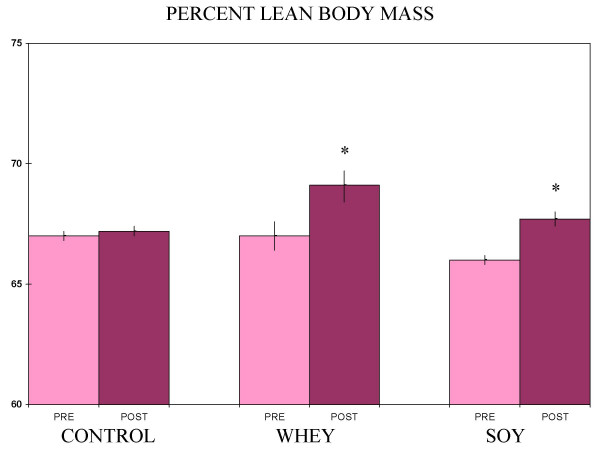
**Lean body mass pre- and post-treatment**. Values are % lean body mass (kg) ± SEM from 9 subjects per group. *Significantly different from pre-treatment values (paired t-test, p < 0.05)

**Figure 2:**
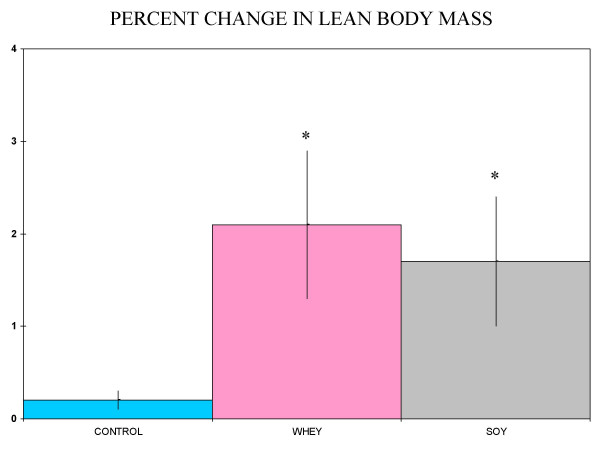
**Percent change lean body mass**. Values are % change in lean body mass ± SEM. *Different letters indicate significantly differences between groups (Tukey test, p < 0.05)

**Figure 3:**
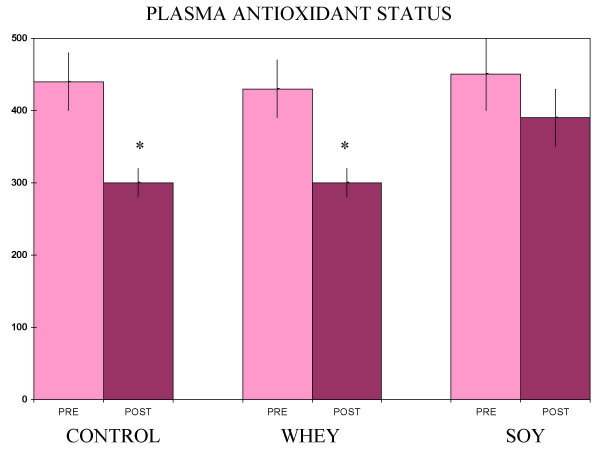
**Plasma antioxidant status**. Values are mM of trolox equivalents ± SEM (N = 5 for control and whey, 8 for soy) *Significantly different from pre-treatment values (paired t-test, p < 0.05)

**Figure 4:**
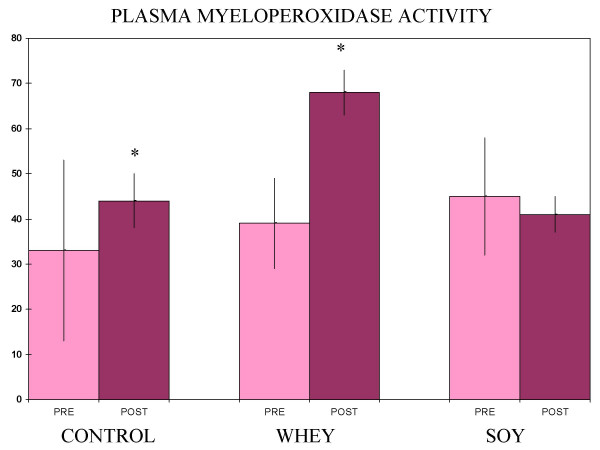
**Plasma myeloperoxidase**. Values are mg/L ± SEM (N = 5 for control and whey, 8 for soy) *Significantly different from pre-treatment values (paired t-test, p < 0.05) **Significantly different from pre-treatment values (paired t-test, p < 0.01)

Baseline subject characteristics are given in Table 1. Exercise training plus soy or whey treatments each produced a statistically significant increase in lean body mass, but the training alone did not (Figure 1). A comparison of the change in lean body mass for the soy group versus the change in the whey group did not show a significant difference (Figure 2). Plasma radical scavenging capacities fell in the whey and training alone groups, while the myeloperoxidase values rose in those same two groups (Figures 3 and 4). The values were unchanged in the soy group (Figures 3 and 4).

**Table 1:** Subject characteristics.

	WHEY	SOY	CONTROL (Training Alone)
AGE	20.36 ± 0.34	21.67 ± 0.24	20.44 ± 0.63
HEIGHT (cm)	180 ± 1.55	179 ± 1.30	178 ± 1.81
WEIGHT (kg)	81 ± 2.81	79 ± 2.49	79 ± 0.48
LBM (kg)	67 ± 1.96	66 ± 2.30	67 ± 1.65

Values are means ± SEM.

## Discussion

In this study, soy and whey were both effective at increasing lean body mass with exercise training, but the soy had the added advantage of inhibiting two negative effects of training on antioxidant status. The percent change in the radical scavenging capacity (total antioxidant status) seen with training alone and training plus whey was substantial compared to the differences typically seen for these types of measurements[11-13].

The lean body mass data seen here contradicts the common, but unconfirmed notion that soy is inferior to whey for promoting lean body mass gain. It should be noted, however, that the general trend for this study may or may not be duplicated for other study designs. For example, the time frame used here, 9 weeks, is not overly long for seeing lean body mass gain, which may explain why the training alone did not produce an effect on lean body mass gain. Thus, the effects of soy or whey on lean body mass gain versus training alone may be more pronounced than in longer studies. It should also be noted that the training program used here emphasized low exercise repetitions in subjects not used to this type of training. In addition, this study included only subjects that were still relatively early in their training experience, and placed no restriction on Calorie intake. These design considerations were geared toward gaining bulk and power. The effects of whey or soy on lean body mass might be different in a design that emphasizes higher repetitions or Calorie restriction in other types of subjects. In addition, it can be noted that the current study diet intervention used bars which included added micronutrients. Thus, this study did not determine if the effects of the soy or whey protein required co-administration of micronutrients.

It is not known whether the negative effects of training seen here for antioxidant status in the whey plus training alone groups would continue upon longer training. The current state of knowledge concerning exercise training effects on antioxidant defenses does not present a clear pattern [i.e. [4,5,8,9]], possibly because of the highly variable circumstances involved in different studies such as training intensity, types of exercise done, types of antioxidant measures used, fitness level of the subjects, length of training, and dietary patterns of the subjects. These variables may help explain why some studies find training-induced declines in antioxidant defense while others find no change or even an increase. Nonetheless, the present study suggests that soy protein intake can promote antioxidant function during training which could be helpful no matter what the effects of training by itself.

Another unresolved issue is whether the effects on lean body mass seen here for the two proteins were due to increased total protein intake or other factors. In regard to the former, the data regarding the amount and type of protein intake necessary to produce optimal strength training gains is conflicting. While a diet meeting the current RDA for protein intake (0.8 g/kg body mass) may be sufficient for the sedentary individual, recent studies suggest dietary protein exceeding that of the RDA is needed for muscle hypertrophy [14,15]. One of the difficulties in deriving an exact protein recommendation for exercisers is that total energy intake has not been consistent in the studies. In some studies, total energy intake was low, which can cause an abnormally high percentage of energy output to be derived from protein [15,16]. In the present study, a 3 day diet record gave no indication that Calorie intake was low (data not shown).

If soy and whey promotion of lean body mass gain was not due to increased total protein intake, which remains uncertain, then other factors were responsible. In the case of soy protein, there are associated antioxidants [2]. As presented in the Introduction, this could conceivably help indirectly with lean body mass gain. In the case of whey, the content of essential amino acids, especially those with sulfur, may be conducive to promoting lean body mass gain [i.e. [17,18]].

In summary, soy and whey protein bars both supported lean body mass gain in conjunction with a short term power-based weight training program, but only the soy bar prevented a training-induced drop in antioxidant capacities.

## Competing interests

Author AB owns the company that produces the soy bars used in the study.

## Authors' contributions

ECB planned and carried out specifics of the intervention. RAD conceived the general aims of the study and chose the blood measurements. AB invented the protein bars and planned specifics of the nutrition intervention. STD planned the general aspects of the exercise intervention.
